# Gut Microbiota and Type 2 Diabetes: Genetic Associations, Biological Mechanisms, Drug Repurposing, and Diagnostic Modeling

**DOI:** 10.3390/ijms27021070

**Published:** 2026-01-21

**Authors:** Xinqi Jin, Xuanyi Chen, Heshan Chen, Xiaojuan Hong

**Affiliations:** School of Acupuncture–Moxibustion and Tuina, Chengdu University of Traditional Chinese Medicine, Chengdu 611137, China; jinxinqi@stu.cdutcm.edu.cn (X.J.); chenxuanyi@stu.cdutcm.edu.cn (X.C.); chs2631569797@163.com (H.C.)

**Keywords:** type 2 diabetes, gut microbiota, Mendelian randomization, network pharmacology, diagnostic modeling

## Abstract

Gut microbiota is a potential therapeutic target for type 2 diabetes (T2D), but its role remains unclear. Investigating causal associations between them could further our understanding of their biological and clinical significance. A two-sample Mendelian randomization (MR) analysis was conducted to assess the causal relationship between gut microbiota and T2D. Key genes and mechanisms were identified through the integration of Genome-Wide Association Studies (GWAS) and cis-expression quantitative trait loci (cis-eQTL) data. Network pharmacology was applied to identify potential drugs and targets. Additionally, gut microbiota community analysis and machine learning models were used to construct a diagnostic model for T2D. MR analysis identified 17 gut microbiota taxa associated with T2D, with three showing significant associations: *Actinomyces* (odds ratio [OR] = 1.106; 95% confidence interval [CI]: 1.06–1.15; *p* < 0.01; adjusted *p*-value [*p*_adj_] = 0.0003), *Ruminococcaceae* (UCG010 group) (OR = 0.897; 95% CI: 0.85–0.95; *p* < 0.01; *p*_adj_ = 0.018), and *Deltaproteobacteria* (OR = 1.072; 95% CI: 1.03–1.12; *p* < 0.01; *p*_adj_ = 0.029). Ten key genes, such as *EXOC4* and *IGF1R*, were linked to T2D risk. Network pharmacology identified *INSR* and *ESR1* as target driver genes, with drugs like Dienestrol showing promise. Gut microbiota analysis revealed reduced α-diversity in T2D patients (*p* < 0.05), and β-diversity showed microbial community differences (R^2^ = 0.012, *p* = 0.001). Furthermore, molecular docking confirmed the binding affinity of potential therapeutic agents to their targets. Finally, we developed a class-weight optimized Extreme Gradient Boosting (XGBoost) diagnostic model, which achieved an area under the curve (AUC) of 0.84 with balanced sensitivity (95.1%) and specificity (83.8%). Integrating machine learning predictions with MR causal inference highlighted *Bacteroides* as a key biomarker. Our findings elucidate the gut microbiota-T2D causal axis, identify therapeutic targets, and provide a robust tool for precision diagnosis.

## 1. Introduction

Diabetes mellitus (DM) is an endocrine–metabolic disease characterized by chronically elevated blood glucose levels [1,2]. It is frequently accompanied by complications affecting the heart, eyes, kidneys, and nervous system. The disability and mortality rates of DM are increasing year by year [3]. In 2021, approximately 521 million individuals worldwide were diagnosed with diabetes, 96% of whom had type 2 diabetes (T2D) [3]. Currently, T2D treatments primarily focus on managing hyperglycemia through oral hypoglycemic drugs, insulin therapy, and lifestyle modifications rather than addressing the underlying metabolic disturbances [4]. Blood glucose testing remains the primary diagnostic tool [5]; however, it only reflects abnormalities in glucose metabolism on a superficial level and cannot directly address the underlying metabolic dysregulations that drive these outcomes. The significant individual variations in insulin resistance, β-cell dysfunction, and other metabolic abnormalities, along with the differing degrees and sequences of these factors in T2D patients, complicate the development of a standardized treatment approach [6]. Consequently, early warning and personalized treatment for diabetes remain inadequate. There is an urgent need to explore precision medicine strategies that address the root causes of metabolic dysregulation to improve treatment efficacy and reduce complication rates [7,8].

Studies indicate significant structural differences in gut microbiota between healthy individuals and T2D patients, suggesting a potential role in the onset and progression of T2D [9,10,11]. The gut microbiota, regarded as a “second genome” [9], participates in physiological processes such as digestion, immunity, and metabolism, influencing T2D progression by regulating gene expression [12]. Furthermore, it modulates drug metabolism and efficacy through its interactions with pharmaceuticals [13], impacting aspects like bioavailability, biotransformation, distribution, elimination, and potential toxicity [14,15]. Therefore, in-depth analysis of the gut microbiota could reveal new drug targets, enable more personalized treatments, and identify potential biomarkers for developing gut microbiota-based diagnostic models to achieve more precise treatment strategies.

Currently, recent advancements in Genome-Wide Association Studies (GWAS) have enhanced our understanding of T2D’s genetic architecture [16]. Mendelian randomization (MR) analysis, which uses genetic variants as instrumental variables (IVs), provides a powerful method for exploring causal relationships between the gut microbiome and T2D [17]. The network pharmacology approach offers a novel framework for constructing drug–target–gene networks, aimed at drug repurposing [18]. Additionally, identifying clinical features of T2D and integrating machine learning methods allows for the development of gut microbiota-based diagnostic models for T2D, enhancing early diagnostic accuracy and offering new insights into personalized treatment. The aim of the present work was fourfold: (a) clarify the causal relationship between gut microbiota and T2D; (b) identify key genes and biological mechanisms linking gut microbiota and T2D; (c) screen for effective drugs and predict potential new uses; and (d) construct diagnostic models by identifying clinical features of T2D from gut microbiota metagenomic classification data.

## 2. Results

### 2.1. Mendelian Randomisation

**Figure 1:**
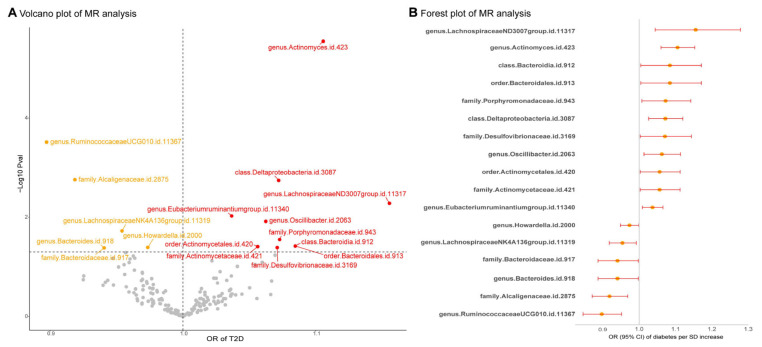
Result summary of MR analysis on the associations between gut microbiota and the risk of T2D: (**A**) This volcano plot illustrates the causal associations between gut microbiota and T2D based on MR analysis. The *x*-axis represents the OR, while the *y*-axis indicates the −log10(*p*-value). Red and orange dots signify gut microbial taxa significantly positively and negatively associated with T2D, respectively, while gray dots represent taxa that did not reach statistical significance. (**B**) This forest plot presents the OR and 95% CI for 17 gut microbial taxa significantly associated with the risk of T2D based on MR analysis. MR, Mendelian randomization; T2D, type 2 diabetes; OR, odds ratio; CI, confidence interval.

Using Inverse–variance weighted (IVW) MR analysis, we identified several gut microbiota taxa associated with T2D risk (Figure 1 and Tables S1 and S2). Specifically, the *Lachnospiraceae* (NK4A136 group) genus, *Ruminococcaceae* (UCG010 group) genus, *Howardella* genus, *Bacteroides* genus, *Bacteroidaceae* family, and *Alcaligenaceae* family were negatively associated with T2D risk. In contrast, the *Oscillibacter* genus, *Eubacterium* (ruminantium group) genus, *Actinomyces* genus, *Lachnospiraceae* (ND3007 group) genus, *Desulfovibrionaceae* family, *Actinomycetaceae* family, *Porphyromonadaceae* family, *Actinomycetales* order, *Bacteroidales* order, *Deltaproteobacteria* class, and *Bacteroidia* class were positively associated with T2D risk. After adjusting for the false discovery rate (FDR), only the *Actinomyces* genus, *Ruminococcaceae* (UCG010 group) genus, and *Deltaproteobacteria* class remained statistically significant (*p*_adj_ < 0.05). Cochran’s Q test indicated significant heterogeneity in several associations, prompting the use of random-effects MR estimates. No evidence of horizontal pleiotropy was found, and the leave-one-out sensitivity analysis confirmed the robustness of our findings (Tables S3–S6 and Figures S1 and S2). Finally, reverse MR analysis revealed no causal relationship between these gut microbiota taxa and T2D outcomes (Tables S7–S12 and Figures S3 and S4).

### 2.2. Genes and Functions

**Figure 2:**
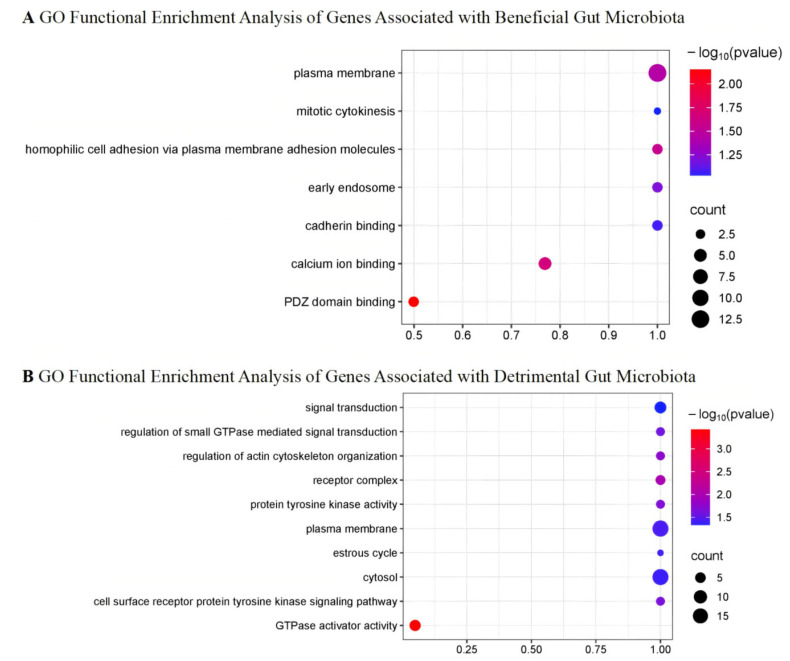
GO terms for potential disease-related key genes associated with gut microbiota: (**A**) This figure shows the GO functional enrichment results of genes associated with beneficial gut microbiota (OR < 1). (**B**) This figure shows the GO functional enrichment results of genes associated with detrimental gut microbiota (OR > 1). GO, Gene Ontology; OR, odds ratio.

The relationships between Single nucleotide polymorphisms (SNPs), genes, and their associated functions are detailed in Tables S13 and S14. Gene Ontology (GO) analysis indicates that gut microbiota linked to favorable disease outcomes may be involved in processes such as PDZ domain binding, calcium ion binding, homophilic cell adhesion via plasma membrane adhesion molecules, and plasma membrane organization. In contrast, gut microbiota associated with unfavorable disease outcomes may participate in processes such as GTPase activator activity, receptor complex formation, regulation of actin cytoskeleton organization, protein tyrosine kinase activity, cell surface receptor protein tyrosine kinase signaling, regulation of small GTPase-mediated signal transduction, plasma membrane dynamics, estrous cycle, cytosolic functions, and signal transduction pathways (Figure 2). No significant enrichment of pathways was identified in the Kyoto Encyclopedia of Genes and Genomes (KEGG) pathway analysis. Through Summary-data-based Mendelian randomization (SMR) and Heterogeneity in Dependent Instrumental Variables (HEIDI) analysis, the genes *EXOC4*, *IGF1R*, *DHX32*, *USP35*, *MRVI1*, *ARRDC4*, *ADNP*, *BCCIP*, *RAP1GAP2*, and *AUTS2*, mapped by these IVs, were found to be significantly associated with T2D (*p*_SMR_ < 0.05, *p*_HEIDI_ > 0.05) (Tables S15 and S16 and Figure S5).

### 2.3. Network Pharmacology Predicts Related Drugs

**Figure 3:**
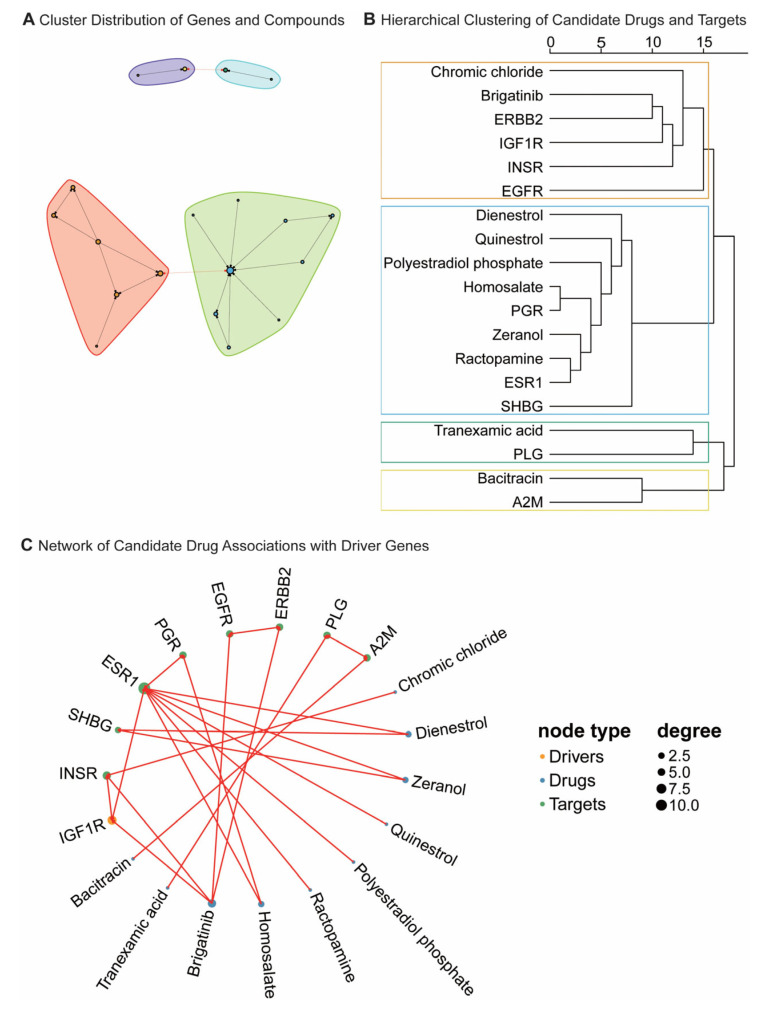
Network pharmacology-based analysis of drug–target–driver gene composite regulatory networks: (**A**) This figure shows the clustering of biological entities into functional groups, or “cliques,” based on potential interactions and pathway relationships. Each cluster contains nodes that likely participate in similar BP or pathways, highlighting key relationships within biological systems. Colored regions demarcate distinct functional modules representing sex hormone regulation (red), cell growth and cancer therapy (green), hemostasis mechanisms (purple), and protease defense (cyan). (**B**) The dendrogram illustrates the hierarchical clustering relationships between various compounds and their target receptors. The horizontal axis represents the relative distance or similarity in clustering, with closer nodes indicating higher molecular structural or biological activity similarity between compounds and targets. Colored boxes demarcate distinct functional modules representing cell growth and cancer therapy (orange), sex hormone regulation (blue), hemostasis mechanisms (green), and protease defense (pale yellow). (**C**) This figure displays the associations between candidate drugs and driver genes, with red lines indicating significant associations between candidate drugs and corresponding driver genes. Node size represents the degree of connectivity of genes within the network. BP, biological processes.

Network pharmacology identified a total of 1874 drugs that target driver genes (Table S17), with 86 exhibiting statistically significant differences (*p* < 0.05), indicating their potential therapeutic efficacy in the treatment of diabetes. Among these, the four drugs with the highest affinities were Chromic chloride, Dienestrol, Zeranol, and Quinestrol (Figure 3), with a proximity score of −2.932, demonstrating strong associations with key genes such as *INSR*, *SHBG*, and *ESR1*. Based on Absorption, Distribution, Metabolism, Excretion, and Toxicity (ADMET) analysis, Dienestrol, Quinestrol, and Zeranol exhibited distinct pharmacokinetic and toxicity profiles, as detailed in Supplementary Tables S18 and S19.

### 2.4. Gut Microbiota Diversity and Structural Differences

**Figure 4:**
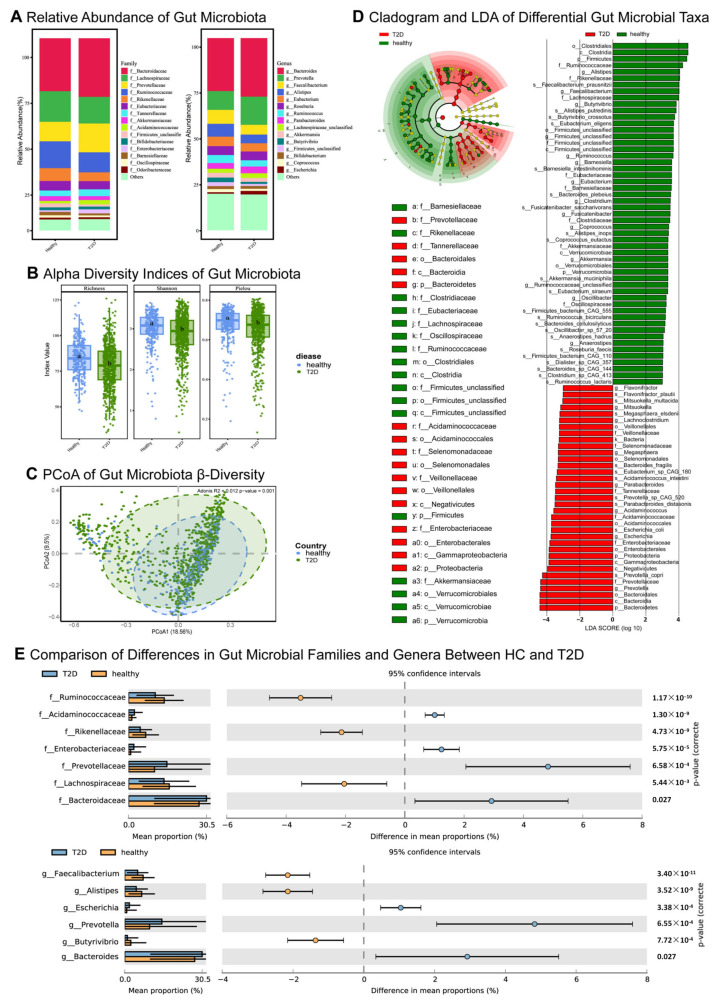
Analysis of gut microbiota diversity and structural differences between T2D patients and HC: (**A**) This figure illustrates the differences in gut microbiota composition and diversity between T2D patients and HC, showcasing variations in microbial populations between the two groups. (**B**) This figure shows the alpha diversity indices (Richness, Shannon, and Pielou), indicating that microbial diversity is significantly higher in the healthy group compared to the T2D group. Different lowercase letters (a, b) indicate statistically significant differences between the groups (*p* < 0.05). (**C**) This figure presents the beta diversity differences between the two groups through PCoA, which reveals significant variations in microbial community structure between T2D patients and HC (R^2^ = 0.012, *p* = 0.001). (**D**) This figure highlights microbial taxa with significant differences at the family and genus levels, where red denotes taxa with higher abundance in the T2D group and green denotes taxa with higher abundance in the healthy group. (**E**) This figure shows the differences in microbial abundance between HC and T2D patients at the family and genus levels. The left side of each panel displays the mean proportion of different microbial taxa in both groups, while the right side shows the 95% CI for the differences in mean proportions. Blue represents the T2D group, and orange represents the healthy group. T2D, type 2 diabetes; HC, healthy controls; PCoA, principal coordinates analysis; CI, confidence interval.

This study conducted a comparative analysis of the gut microbiota between T2D patients and healthy controls (HC), uncovering substantial differences in microbial composition across various taxonomic levels, including family and genus (Figure 4A). Alpha diversity analysis revealed that microbial diversity in the healthy group was significantly higher than in the T2D group (*p* < 0.05), indicating a more diverse and balanced microbiota in healthy individuals (Figure 4B), whereas T2D patients exhibited reduced diversity and diminished evenness. To further evaluate structural disparities in the microbial communities between the two groups, Principal coordinates analysis (PCoA) was employed. Beta diversity analysis demonstrated significant differences in gut microbiota composition between T2D patients and HC (R^2^ = 0.012, *p* = 0.001), a finding corroborated by Adonis analysis (Figure 4C). Using the Linear discriminant analysis effect size (LEfSe) method, we identified 88 significantly different microbial taxa. Statistical Analysis of Metagenomic Profiles (STAMP) analysis revealed seven differential taxa at the family level and six at the genus level (Figure 4D,E).

### 2.5. Results of Machine Learning

**Figure 5:**
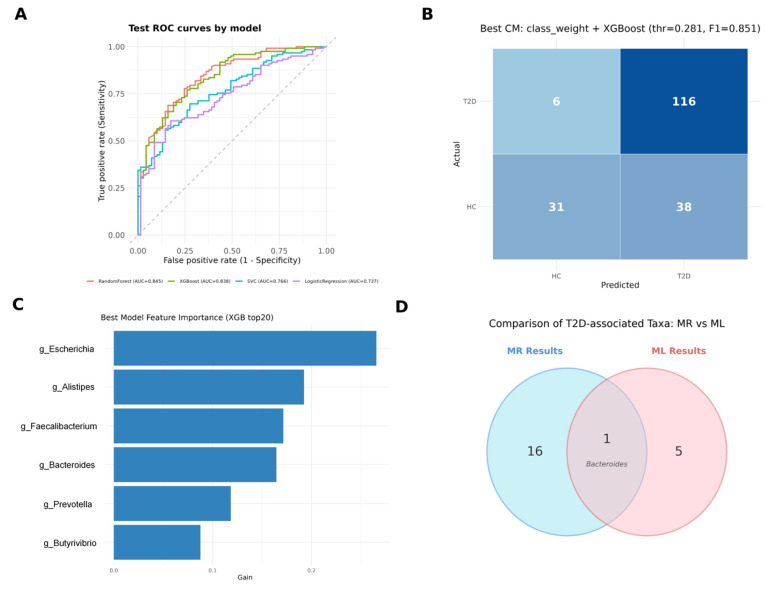
Comprehensive comparison of MR and ML analysis results for T2D-associated gut microbiota: (**A**) ROC curves display the predictive performance of four ML models (Random Forest, XGBoost, SVC, and Logistic Regression) on the test dataset. Random Forest and XGBoost achieved the highest AUC values of 0.845 and 0.838, respectively, while SVC and Logistic Regression showed AUCs of 0.766 and 0.737, demonstrating that ensemble methods outperformed traditional models in T2D prediction. (**B**) The confusion matrix illustrates the performance of the best model (XGBoost with class_weight adjustment, threshold = 0.281, F1 = 0.851) in classifying T2D patients and HC. The model achieved high sensitivity (0.95) for T2D detection with 116 true positives and only 6 false negatives, while showing moderate specificity (0.55) for HC with 38 true negatives and 31 false positives. (**C**) Feature importance plot derived from the XGBoost model reveals the top 20 gut microbiota genera ranked by gain values. (**D**) Venn diagram illustrates the concordance between MR and ML approaches in identifying T2D-associated taxa. MR analysis identified 17 taxa with causal associations to T2D risk, while ML feature selection identified 6 key genera. Bacteroides emerged as the single taxon identified by both methods, with 16 taxa unique to MR and 5 genera unique to ML, demonstrating complementary insights from causal inference and predictive modeling approaches. MR, Mendelian randomization; ML, machine learning; T2D, type 2 diabetes; ROC, receiver operating characteristic; SVC, support vector classification; AUC, area under the curve; HC, healthy controls.

On the held-out test set, Random Forest (RF) and Extreme Gradient Boosting (XGBoost) demonstrated the strongest discriminative performance, achieving an area under the curve (AUC) of 0.845 and 0.838, respectively, outperforming support vector classification (SVC) (AUC = 0.766) and Logistic Regression (LR) (AUC = 0.737) (Figure 5A). The initial classification results suggested a sensitivity–specificity imbalance, with an elevated false-positive rate among HC. After optimization, the class-weight-adjusted XGBoost model provided the best overall balance, yielding the highest F1-score (0.851) with a robust AUC of 0.838. Using the optimized threshold (0.281), the confusion matrix (Figure 5B, Tables S20–S29) indicated improved identification of HC while maintaining high sensitivity: 116/122 T2D cases were correctly classified (recall = 95.1%) and 31/37 controls were correctly classified (specificity = 83.8%).

Model interpretability analyses highlighted Escherichia, Alistipes, Faecalibacterium, Bacteroides, Prevotella, and Butyrivibrio as the most influential genera driving predictions based on mean absolute SHapley Additive exPlanations (SHAP) values (Figure 5C). Cross-validation against MR findings further showed convergence on Bacteroides (Figure 5D), supporting its prioritization as a reproducible predictor and a biologically plausible candidate consistent with a potential causal role in T2D pathogenesis.

## 3. Discussion

In this study, we conducted a two-sample MR analysis using summary statistics from the MiBioGen Consortium’s meta-analysis of gut microbiota and T2D GWAS to investigate the causal association between gut microbiota and T2D. To clarify the intricate association between gut microbiota and T2D, we also employed bidirectional MR analysis, which enabled us to evaluate the interplay between gut microbiota and T2D within a unified analytical framework, unveiling potential underlying biological mechanisms. Furthermore, bidirectional MR is instrumental in systematically addressing reverse causality, which is critical for establishing true causal links and identifying microbiota changes attributable to T2D. By accounting for shared genetic factors, we effectively mitigated confounding variables, thereby strengthening the robustness of our causal inferences. Compared to previous similar studies [19,20], we enhanced the statistical power and generalizability of our findings by incorporating data from multiple cohorts, rendering our results more robust and representative.

We identified several gut microbial taxa with potential protective effects against T2D, including the *Lachnospiraceae* (NK4A136 group), *Ruminococcaceae* (UCG010 group), and *Bacteroides* genera. Conversely, some taxa may have harmful effects, such as the *Oscillibacter*, *Eubacterium* (ruminantium group), *Deltaproteobacteria*, and *Actinomyces* genera, among others. Notably, after false discovery rate correction, three taxa remained statistically robust: *Actinomyces* and *Deltaproteobacteria* were associated with increased risk, while *Ruminococcaceae* (UCG010 group) exhibited a protective direction. These prioritized signals help reduce over-interpretation of nominal associations, and the overall pattern was supported by our sensitivity analyses (including heterogeneity/pleiotropy assessment and leave-one-out analyses) and bidirectional testing.

*Lachnospiraceae*, *Howardella*, *Ruminococcaceae*, and *Bacteroides* are anaerobic bacteria [21], and mounting evidence suggests they play a crucial role in modulating gut inflammation. These taxa are involved in the metabolism of various carbohydrates, producing short-chain fatty acids (SCFAs) [22,23,24]. SCFAs are vital energy sources for colonocytes [25], and the absence of these compounds may lead to functional impairments of the colonic mucosa, resulting in a damaged gut barrier [26]. A damaged gut barrier increases intestinal permeability, allowing dietary antigens and immunostimulants to enter the bloodstream, thereby exacerbating systemic inflammation and promoting autoimmune processes that ultimately contribute to pancreatic β-cell destruction [27]. Moreover, SCFAs bind to G-protein-coupled receptors 43 (GPR43) and 41 (GPR41), stimulating the release of glucagon-like peptide-1 (GLP-1) from L-cells in the distal ileum and colon. GLP-1, a key incretin, lowers blood glucose through multiple mechanisms. It not only promotes pancreatic β-cell proliferation and inhibits apoptosis but also enhances glucose-dependent insulin secretion. Additionally, GLP-1 suppresses appetite and plays a role in lipid metabolism. Studies have shown that increased abundance of *Lachnospiraceae* (NK4A136 group), *Howardella*, *Ruminococcaceae* (UCG010 group), and *Bacteroides* is associated with higher GLP-1 levels, suggesting these taxa may enhance SCFA production and promote GLP-1 secretion, benefiting T2D treatment [28,29].

For *Bacteroides*, several studies suggest that specific *Bacteroides* taxa may positively influence host bile acid metabolism and modulate signaling pathways that intersect with glucose homeostasis, such as Takeda G-protein-coupled receptor 5 (TGR5)/Farnesoid X Receptor (FXR)-mediated pathways, providing a mechanism by which these microbes may exert protective effects in metabolic disorders, including T2D [30].

In contrast, taxa such as *Deltaproteobacteria*, which include sulfate-reducing bacteria, may contribute to metabolic inflammation through production of hydrogen sulfide and other pro-inflammatory metabolites [31], potentially disrupting intestinal barrier function and promoting systemic low-grade inflammation—a recognized driver of insulin resistance. Similarly, *Actinomyces*, although historically characterized in mucosal niches, has been reported to be enriched in diabetic subgingival and gut microbiota surveys, often in the context of dysbiotic, inflammation-prone community structures [32]. The emergence of *Actinomyces* in MR analyses may reflect systemic ecological shifts linked to chronic hyperglycemia and immune perturbations observed in T2D populations [33,34,35]. Other nominally significant taxa, such as *Oscillibacter* and *Eubacterium* (ruminantium group), have been implicated in inflammatory phenotypes or trimethylamine-related pathways associated with cardiometabolic complications [32,36]. Further research is needed to clarify the roles of these bacteria in diabetes pathogenesis.

GO analysis showed that gut microbiota associated with favorable outcomes may be involved in processes like PDZ domain binding, calcium ion binding, cell adhesion, and plasma membrane organization. These processes are key to intercellular signaling, cell adhesion, and cellular homeostasis, suggesting that protective microbiota may promote metabolic balance by enhancing gut barrier function and modulating calcium signaling. The PDZ domain regulates ion channels, cell adhesion, and signal transduction [37], crucial for maintaining intestinal epithelial barrier integrity. Meanwhile, calcium signaling supports this process by promoting calcium influx and modulating adhesion proteins, thereby enhancing tissue stability [38,39]. Consequently, these beneficial microbes may help maintain metabolic homeostasis and prevent invasion by pathogenic microorganisms [39,40]. On the other hand, gut microbiota associated with unfavorable outcomes may be involved in biological processes (BP), such as GTPase activator activity, receptor complex, regulation of actin cytoskeleton organization, and protein tyrosine kinase activity. Studies show that GTPase activator activity linked to gut microbiota affects actin cytoskeleton regulation, influencing cell migration, signal transduction, and inflammation, thus contributing to T2D progression [41]. Rho GTPases impact insulin signaling and glucose metabolism [41]. Protein tyrosine kinases, like Src family kinases, regulate cell adhesion and immune responses, affecting inflammation and T2D-related metabolic disorders [42]. While GO analysis identified functional enrichments, KEGG pathway analysis did not, suggesting that the gut microbiota affects T2D through specific gene functions. Future research should integrate multi-omics data to explore these mechanisms and identify targets for personalized therapies.

SMR and HEIDI prioritization implicated genes such as *EXOC4*, *IGF1R*, *DHX32*, *USP35*, and others in the gut microbiota–T2D axis. While these findings highlight shared genetic associations, the precise causal architecture warrants careful interpretation. Our analysis identifies host genetic variants with pleiotropic effects on both gene expression and T2D susceptibility. These variants may influence T2D risk directly through host molecular pathways, potentially independent of the gut microbiota, or they may modulate the host environment to shape microbial composition. To aid interpretation and generate testable hypotheses, we further leveraged network pharmacology, which yielded a large set of predicted compound–target links. Although 86 compounds reached nominal significance (*p* < 0.05), such signals should be regarded as hypothesis-generating under the multiple-testing burden and, on their own, do not justify clinical repurposing claims. Instead, their primary value here is mechanistic, pointing to endocrine–metabolic crosstalk converging on the *ESR1/SHBG/INSR* axis. Biologically, *ESR1* has been linked to insulin resistance and T2D pathogenesis [43,44,45], and lower *SHBG* has been associated with gestational dysglycemia risk and prediction [46,47,48], and *INSR* remains central to insulin signaling [49,50].

Evidence suggests that chromium supplementation may confer modest improvements in glycemic control and lipid parameters, and multiple experimental and clinical studies have linked chromic chloride to improved insulin sensitivity, glucose tolerance, and partial correction of dyslipidemia—effects more consistent with insulin sensitization than direct insulin secretagogue activity [51,52,53,54]. By contrast, dienestrol, zeranol, quinestrol, and polyestradiol phosphate are estrogenic or estrogen-like compounds. Dienestrol is a synthetic estrogenic compound more often discussed under the broader context that exogenous estrogens can alter glucose tolerance, potentially complicating diabetes management and therefore warranting cautious interpretation of any apparent “metabolic effects” [45,55]. Zeranol, another estrogen-like agent, has been reported to perturb glucose-handling pathways, highlighting endocrine–metabolic crosstalk rather than supporting an antidiabetic therapeutic positioning [56]. For quinestrol, early clinical observations noted glucose-tolerance changes during therapy [57]. For polyestradiol phosphate, available evidence indicates that sustained exogenous estrogen exposure can influence glucose tolerance and lipoprotein profiles, again arguing for cautious interpretation [58]. Finally, ractopamine evidence largely comes from animal production physiology and provides limited support for direct inference regarding diabetes clinical outcomes [59].

Against this backdrop, the most proximal compounds (dienestrol, zeranol, and quinestrol) were prioritized for downstream ADMET assessment to contextualize feasibility and safety. Consistent with this exploratory framing, ADMET profiling suggested distinct pharmacokinetic and toxicity liabilities for dienestrol, quinestrol, and zeranol; moreover, given their estrogenic or estrogen-like activities, these hits are best interpreted as pathway pointers to the *ESR1/SHBG* axis, with known safety/regulatory considerations necessitating careful qualification and experimental validation, alongside prioritization of safer pathway-level alternatives before any translational inference. Additionally, leveraging metagenomic sequencing and machine learning, we further uncovered significant alterations in gut microbiota diversity and composition between T2D patients and HC and constructed a predictive model based on these differences. The significantly lower alpha diversity in T2D patients compared to HC supports previous findings on gut microbiota dysbiosis in T2D pathogenesis [60,61]. Beta diversity analysis showed significant structural differences between T2D and healthy control microbiota, confirmed by Adonis analysis. These shifts may be linked to metabolic dysfunction and chronic inflammation in T2D patients, as gut dysbiosis can influence metabolism and immune responses [62,63]. LEfSe and STAMP analyses identified decreased diversity in T2D, with reduced taxa such as *Faecalibacterium*, *Ruminococcaceae*, and *Lachnospiraceae*, and increased *Acidaminococcaceae* and *Enterobacteriaceae*, suggesting potential biomarkers for T2D.

Leveraging metagenomic sequencing, we constructed a predictive model to distinguish T2D patients from HC. Consistent with ensemble learning advantages, the Random Forest and XGBoost models achieved superior performance, with AUCs of 0.845 and 0.838, respectively [64]. To address the sensitivity–specificity imbalance observed in the initial analysis, we applied a class-weight adjustment strategy to the XGBoost model. This optimization successfully enhanced the model’s specificity to 83.8% while maintaining a high recall of 95.1%, thereby mitigating the false-positive bias common in imbalanced clinical datasets.

Furthermore, we integrated machine learning with MR to bridge the gap between predictive correlation and causal inference. While machine learning (ML) analysis identified high-dimensional discriminative features (e.g., *Escherichia*, *Alistipes*, and *Bacteroides*) based on SHAP values, these associations do not inherently imply causality. By intersecting these top predictive features with causal taxa identified via MR, we pinpointed *Bacteroides* as a convergent biomarker. This triangulation supports *Bacteroides* as a priority target, validated by both its diagnostic robustness and its potential causal role in T2D pathogenesis.

This study has several limitations: (1) MR constraints: While MR analysis reduces confounding factors, unidentified residual confounders may still affect outcomes, and it only shows associations, not causality. Further functional studies are needed to confirm the gut microbiota-T2D causal link. (2) Demographic bias: Both the GWAS summary statistics (MiBioGen) and the metagenomic dataset used for diagnostic modeling were primarily derived from European populations. This geographic and ethnic homogeneity may limit the generalizability of our causal estimates and the diagnostic model’s performance to other ancestries, necessitating validation in diverse, multi-ethnic cohorts. (3) Data interpretation: 16S rRNA sequencing has limited species-level resolution, and relative abundance analysis may introduce bias. Future studies should incorporate absolute abundance and multi-omics approaches. (4) Network pharmacology: Drug target databases are limited, potentially missing candidate drugs, and predictions require experimental validation. (5) Data imbalance: Despite class-weight adjustments, class imbalance may still impact model performance, particularly in classifying the healthy cohort.

In conclusion, we integrated gut microbiota and T2D GWAS data, employing bidirectional MR analysis to elucidate potential causal associations between various gut microbial taxa and T2D. We identified several microbial groups with either protective or detrimental effects and explored the underlying biological mechanisms through GO analysis. Moreover, SMR and HEIDI analyses revealed a set of key genes implicated in both gut microbiota composition and T2D, suggesting that gut microbiota may modulate T2D pathogenesis through the regulation of these genes. Network pharmacology was further utilized to predict potential therapeutic agents. Metagenomic sequencing confirmed significant alterations in gut microbiota diversity and structure between T2D patients and HC. Lastly, we developed a T2D classification model based on microbial features using machine learning, underscoring its potential clinical application value. Our study offers novel insights into the pathogenesis of T2D and lays the foundation for personalized therapeutic strategies.

## 4. Materials and Methods

### 4.1. Study Design

**Figure 6:**
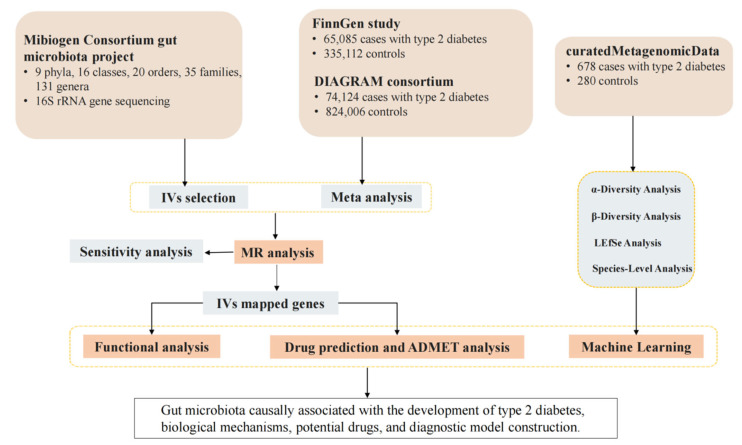
Study design overview.

This study followed the STREGA [65] and STROBE-MR [66] guidelines for MR research. First, a bidirectional two-sample MR analysis was conducted to examine the causal relationship between gut microbiota and T2D. Second, IVs were mapped to genes, and the biological pathways were analyzed to reveal mechanisms linking gut microbiota and T2D. Third, a network pharmacology approach was used to build a drug–target–gene network, screen candidate drugs, and perform ADMET analysis for pharmacokinetics and safety evaluation. Finally, curatedMetagenomicData (cMD) was used to analyze gut microbiota clinical features in T2D patients. Machine learning methods constructed a diagnostic model to improve early T2D diagnosis. An overview of the study design is shown in Figure 6. This study used summarized data and did not require additional ethical clearance.

### 4.2. Data Sources

The genetic data for gut microbiota were obtained from the latest GWAS summary statistics conducted by the MiBioGen Consortium (https://mibiogen.gcc.rug.nl/ (accessed on 20 January 2024)), which included 18,340 participants from 24 cohorts [67]. In this study, 15 microbial taxonomic groups lacking specific species names were excluded, resulting in a total of 196 bacterial taxonomic groups.

The summary dataset for T2D was sourced from the DIAbetes Genetics Replication And Meta-analysis (DIAGRAM) consortium [68] and the FinnGen study [69]. The DIAGRAM consortium includes 32 studies with 74,124 cases and 824,006 controls, while the FinnGen study (R10 data) includes 65,085 cases and 335,112 controls, with no overlap between the datasets. A meta-analysis of these two datasets resulted in 139,209 cases and 1,159,118 controls.

The cis-expression Quantitative Trait Loci (cis-eQTL) summary statistics were derived from the Consortium for the Architecture of Gene Expression (CAGE) study [70], which analyzed transcriptional gene expression in the peripheral blood of 2765 individuals, primarily of European ancestry.

Taxonomic profiles of the gut microbiome in individuals with T2D and HC were obtained from the cMD R package (version 3.0, CUNY Graduate School of Public Health and Health Policy, New York, NY, USA) [71]. The dataset includes stool samples from 678 T2D patients and 280 HC from European cohorts.

### 4.3. MR Analysis

This study applied the MR approach, based on three key assumptions [72]: (1) IVs are strongly associated with gut microbiota; (2) IVs are not linked to unmeasured confounders of T2D; and (3) IVs influence T2D only through gut microbial taxa. In selecting IVs, gut microbiota-related SNPs were identified based on a genome-wide significance threshold (*p* < 1 × 10^−5^) [73]. Linkage disequilibrium (LD) among the selected SNPs was assessed using a clumping procedure (R^2^ < 0.001, clumping distance = 10,000 kb) to ensure that only independent SNPs were retained. Furthermore, the F-statistic was calculated to assess potential bias due to weak IVs, and IVs with F-statistics < 10 were excluded. To further guarantee the validity of the selected IVs, exposures with fewer than three SNPs were also removed from the analysis. Additionally, the GWAS meta-analysis was performed by the METAL software (version 2011-03-25, Center for Statistical Genetics, University of Michigan, Ann Arbor, MI, USA) [74]. Genetic variants associated with T2D at *p* < 5 × 10^−8^ in this GWAS meta-analysis and with low LD (R^2^ < 0.001) were selected as the IV for T2D in the reverse MR analysis.

In the main analysis, we primarily used the IVW method for causal inference. Then, alternative models were applied, including the weighted median, MR–Egger, simple mode, and weighted mode methods. Cochran’s Q was computed to quantify heterogeneity across the individual causal effects, and a leave-one-out sensitivity analysis was conducted to identify potential outliers and assess the robustness of the results. Additionally, we employed the Benjamini–Hochberg (BH) method for FDR correction. Results are considered to show a significant association if both the nominal *p*-value and the *p*_adj_ are less than 0.05; results are considered suggestive of an association if the nominal *p*-value is less than 0.05, but the *p*_adj_ is greater than 0.05. To identify the potential reverse causality between T2D and gut microbiota, a reverse MR analysis was conducted to account for the potential impact of T2D on gut microbial composition. All MR analyses were performed using R software (version 4.4.0, R Foundation for Statistical Computing, Vienna, Austria, https://www.rproject.org/) and the “TwoSampleMR” package (version 0.6.22, MRC Integrative Epidemiology Unit, University of Bristol, Bristol, UK).

### 4.4. SNP-Mapped Genes

We utilized SNPnexus (https://www.snp-nexus.org/v4/ (accessed on 27 January 2024)), a web-based variant annotation tool, to map each variant to its nearest gene, which could be an overlapping gene or one located downstream or upstream of the variant [75]. Based on mapping results, we subsequently conducted SMR analysis using SMR software (version 1.3.1, Westlake University, Hangzhou, Zhejiang, China), integrating cis-eQTL summary statistics from the CAGE dataset and the Genotype-Tissue Expression (GTEx) project (v8). The top associated cis-QTL were selected by passing a *p* threshold of 5 × 10^−8^. All independent *cis*-eQTLs associated with the target genes, with a conditional *p*_SMR_ < 0.05 and *p*_HEIDI_ > 0.05, were included in further analysis [76].

### 4.5. Pathway Enrichment Analysis of Key Genes

In the MR analysis, gut microbiota with an odds ratio (OR) greater than 1 were considered detrimental, whereas an OR less than 1 indicated beneficial gut microbiota. Functional enrichment analysis was conducted for genes mapped by IVs associated with both groups of gut microbiota. Using the DAVID database (https://www.diagram-consortium.org/index.html (accessed on 27 January 2024)), we performed enrichment analysis for BP, cellular components (CC), and molecular functions (MF) in GO and KEGG pathways. A significance threshold was set at *p* < 0.05, and the top 10 most significant GO terms and pathways were visualized using the “ggplot2 (version 3.5.1, Springer-Verlag, New York, NY, USA)” R package.

### 4.6. Network Pharmacology-Based Drug Repurposing

Significantly associated genes were identified as driver genes, and 15,407 FDA-approved drug-target pairs were retrieved from the DrugBank database, along with 116,606 protein interaction pairs from the STRING database. Utilizing the drug proximity algorithm [18], we integrated the human protein–protein interaction (PPI) network to construct a composite regulatory network involving drug–target–driver genes, thereby estimating the correlation between each candidate drug and the disease and ultimately identifying candidate drugs significantly associated with the disease. Furthermore, to predict the clinical efficacy and potential adverse effects of the candidate drugs, we conducted in vivo evaluations of their pharmacokinetics and safety through ADMET analysis (absorption, distribution, metabolism, excretion, and toxicity) [77]. The ADMET analysis was performed using ADMETlab (version 3.0, Central South University, Changsha, Hunan, China) [78] and the TEST software (version 5.1.2, U.S. Environmental Protection Agency, Washington, DC, USA) based on Quantitative Structure-Activity Relationship (QSAR) models (https://www.epa.gov/).

### 4.7. Metagenomic Bacterial Community Analysis

All sample sequences were rarefied to 10,000 sequences per sample, which was sufficient to capture the diversity among samples. Chao1, Shannon, and Pielou indices were selected as α-diversity metrics, and differences in α-diversity between groups were compared using the Mann–Whitney U test. Beta diversity was analyzed through PCoA based on Bray–Curtis distances between samples, and the significance of group differences was assessed using Adonis analysis. The online platform ImageGP (https://www.bic.ac.cn/BIC/#/ (accessed on 10 February 2024)) was used for LEfSe analysis, with non-parametric Kruskal–Wallis rank-sum tests identifying species with significant differences in abundance between groups, followed by Wilcoxon rank-sum tests to evaluate consistency of these differential species across subgroups. Linear discriminant analysis (LDA) was then applied to estimate the impact of each species’ abundance on group differences, with an LDA score cutoff of 3. Species-level differences were analyzed using the Stamp software (version 2.1.3, Dalhousie University, Halifax, NS, Canada), and only species with *p* < 0.05 (Welch’s *t*-test, FDR) were shown and considered for composition analysis. Bacterial community analysis and visualization were performed using the R packages phyloseq (version 1.54.0, Stanford University, Stanford, CA, USA), vegan (version 2.6–10, University of Oulu, Oulu, Finland), ggplot2 (version 3.5.1, Springer-Verlag, New York, NY, USA), tidyverse (version 2.0.0, Posit Software, PBC, Boston, MA, USA), tidyr (version 1.3.1, Posit Software, PBC, Boston, MA, USA), agricolae (version 1.3-7, Universidad Nacional Agraria La Molina, Lima, Peru), FSA (version 0.10.0, Northland College, Ashland, WI, USA), microeco (version 1.16.0, Fujian Agriculture and Forestry University, Fuzhou, Fujian, China), and VennDiagram (version 1.7.3, Ontario Institute for Cancer Research, Toronto, ON, Canada).

### 4.8. Development of Machine Learning-Based Diagnostic Model

We incorporated the differential genera as key features and partitioned the dataset into an 80% training set and a 20% testing set [79]. Four ML algorithms were evaluated: RF, SVC, LR, and XGBoost. To address class imbalance, we conducted a systematic ablation study comparing random undersampling, SMOTE, and class-weight adjustment strategies against a baseline model. Hyperparameter optimization was performed using 10-fold cross-validation, and the optimal classification threshold was determined by maximizing the F1-score based on out-of-fold (OOF) predictions. The final model performance was assessed on the independent test set using Receiver operating characteristic (ROC)-AUC, precision-recall metrics, and confusion matrices. To interpret model decisions, we calculated SHAP values using shapviz (version 0.10.3, La Mobilière, Bern, Switzerland) and kernelshap (version 0.9.1, La Mobilière, Bern, Switzerland) packages, visualizing feature contributions via beeswarm and dependence plots. Finally, top predictive features were cross-validated with MR results to confirm biological consistency. All analyses were conducted in R.

## Data Availability

The original contributions presented in this study are included in the article/Supplementary Materials. Further inquiries can be directed to the corresponding author.

## Supplementary Materials

The following supporting information can be downloaded at: https://www.mdpi.com/article/10.3390/ijms27021070/s1.

## Abbreviations

DM	Diabetes mellitus
T2D	Type 2 diabetes
GWAS	Genome–Wide Association Studies
MR	Mendelian randomization
IVs	Instrumental variables
IVW	Inverse–variance weighted
FDR	False discovery rate
SNPs	Single nucleotide polymorphisms
GO	Gene Ontology
KEGG	Kyoto Encyclopedia of Genes and Genomes
SMR	Summary-data-based Mendelian randomization
HEIDI	Heterogeneity in dependent instrumental variables
ADMET	Absorption, Distribution, Metabolism, Excretion, and Toxicity
HC	Healthy controls
PCoA	Principal coordinates analysis
LEfSe	Linear discriminant analysis effect size
STAMP	Statistical Analysis of Metagenomic Profiles
RF	Random forest
XGBoost	Extreme Gradient Boosting
AUC	Area under the curve
SVC	Support vector classification
LR	Logistic regression
SHAP	SHapley Additive exPlanations
SCFAs	Short-chain fatty acids
GPR	G-protein-coupled receptor
GLP-1	Glucagon-like peptide-1
TGR5	Takeda G-protein-coupled receptor 5
FXR	Farnesoid X Receptor
BP	Biological processes
ML	machine learning
cMD	curatedMetagenomicData
DIAGRAM	DIAbetes Genetics Replication And Meta-analysis
cis-eQTL	cis-expression Quantitative Trait Loci
CAGE	Consortium for the Architecture of Gene Expression
LD	Linkage disequilibrium
BH	Benjamini–Hochberg
GTEx	Genotype-Tissue Expression
OR	Odds ratio
CC	Cellular components
MF	Molecular functions
PPI	Protein–protein interaction
QSAR	Quantitative Structure-Activity Relationship
LDA	Linear discriminant analysis
OOF	Out-of-fold
ROC	Receiver operating characteristic
