# Supplementary material for: Gut Microbiota and Type 2 Diabetes: Genetic Associations, Biological Mechanisms, Drug Repurposing, and Diagnostic Modeling
Source: Int J Mol Sci. 2026 Jan 21;27(2):1070. doi: 10.3390/ijms27021070 (PMC12842411; doi:10.3390/ijms27021070)
Supplement: Supplementary file 1 [file ijms-27-01070-s001.zip › supplementary material_Figures/supplementary material_figS1.pdf]

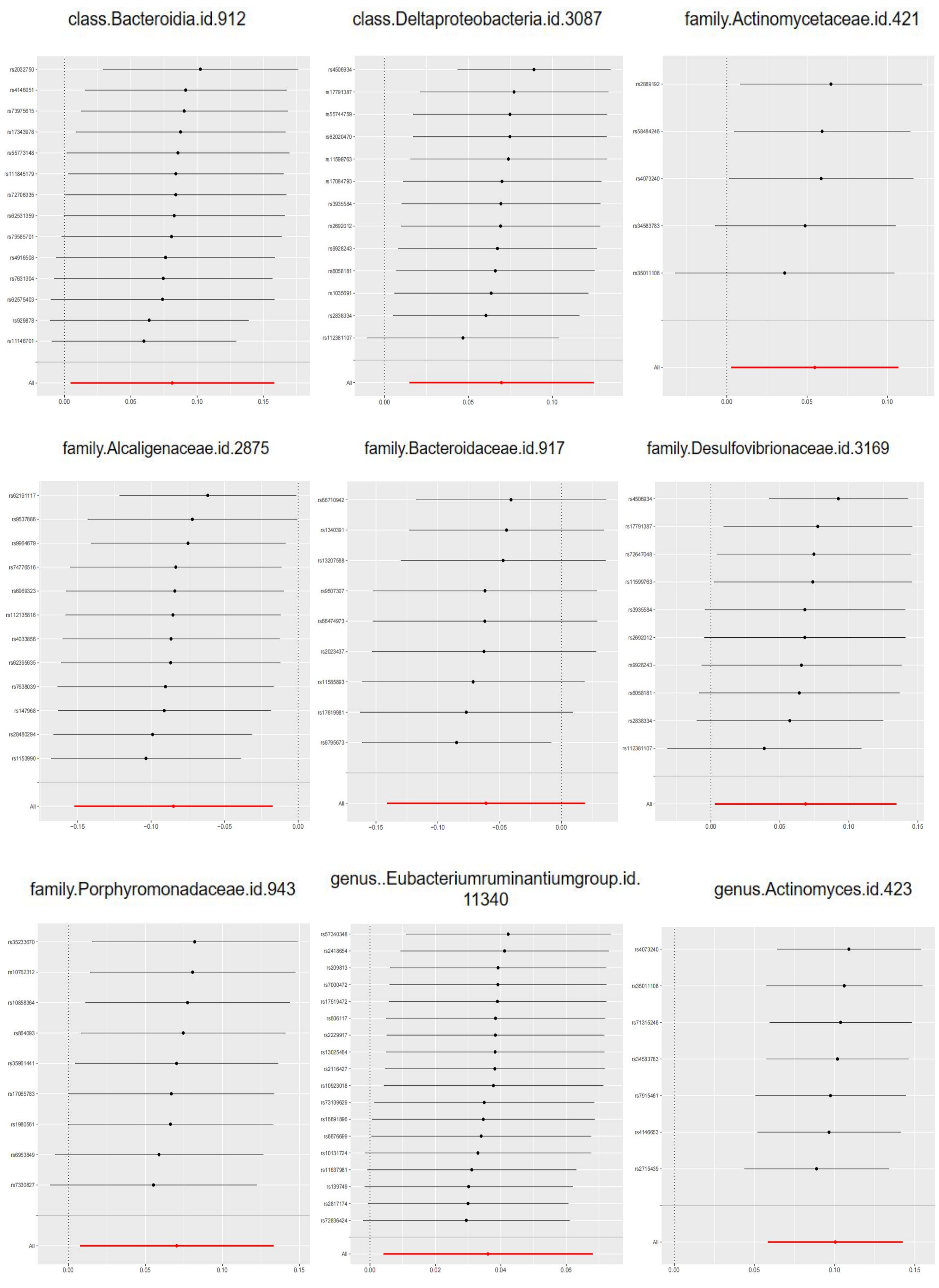

Figure S1 (page 1 of 2). Legend is provided on page 2.

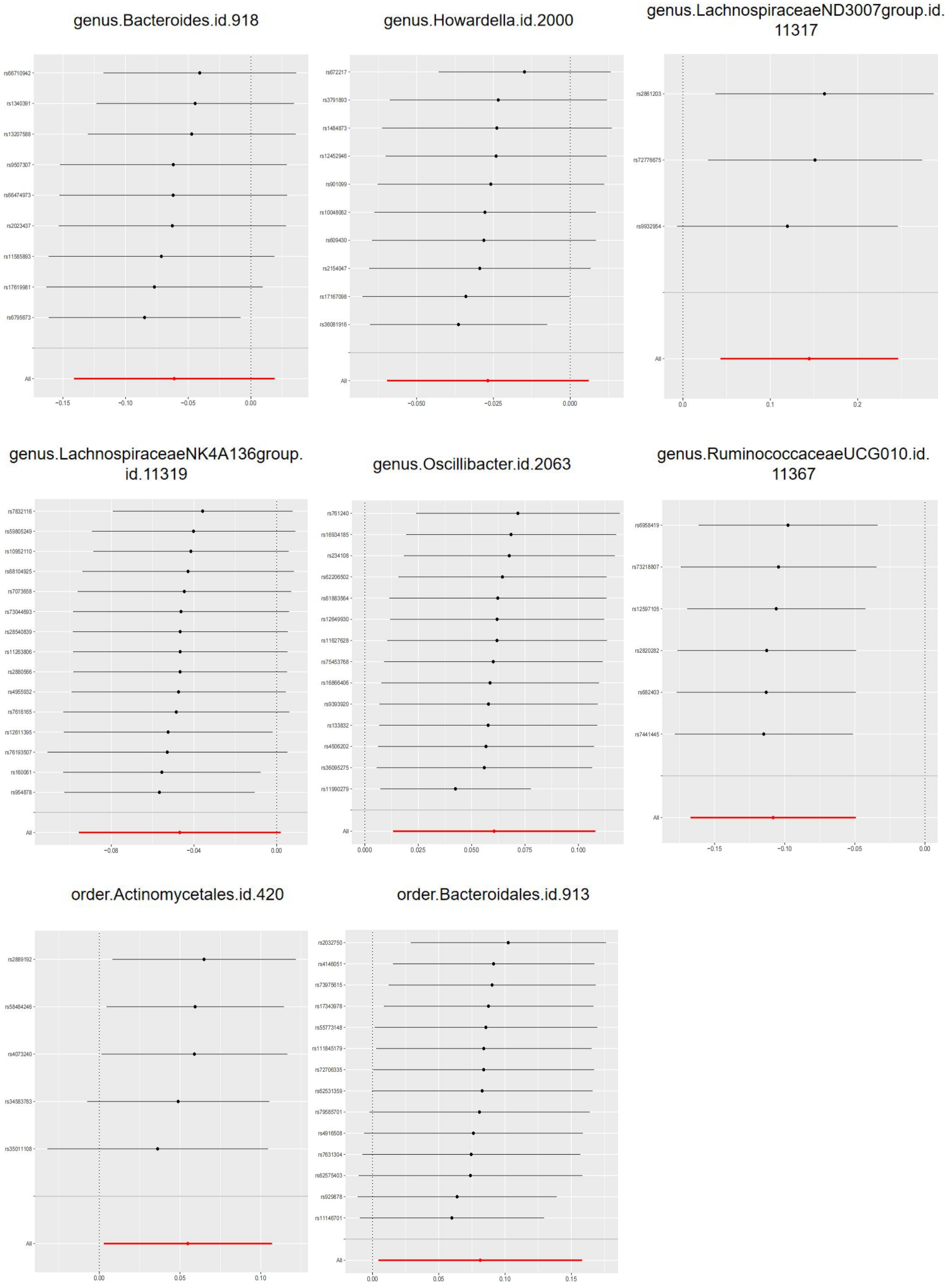

Figure S1. Leave–one–out plots for the causal association between gut microbiota and T2D. Each panel shows leave–one–out MR results, where the causal effect estimate is recalculated after removing one SNP instrument at a time (rsID listed on the y–axis). Black points indicate the MR point estimates and horizontal lines represent 95% confidence intervals. The red "All" row denotes the overall MR estimate using all included SNP instruments. Consistency of the leave–one–out estimates with the "All" estimate suggests that the MR result is not driven by a single SNP.
