# Supplementary material for: Gut Microbiota and Type 2 Diabetes: Genetic Associations, Biological Mechanisms, Drug Repurposing, and Diagnostic Modeling
Source: Int J Mol Sci. 2026 Jan 21;27(2):1070. doi: 10.3390/ijms27021070 (PMC12842411; doi:10.3390/ijms27021070)
Supplement: Supplementary file 1 [file ijms-27-01070-s001.zip › supplementary material_Figures/supplementary material_figS3.pdf]

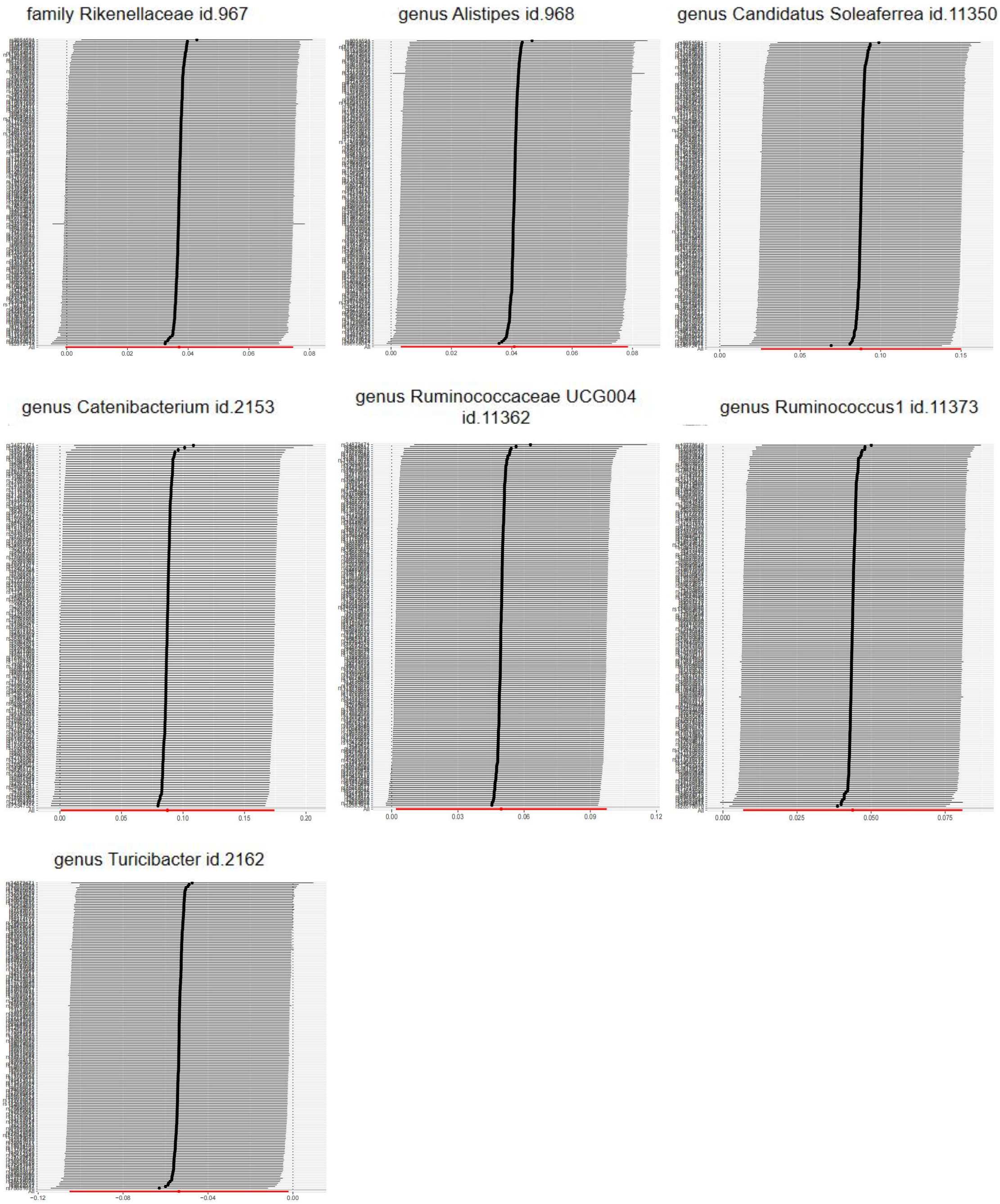

Figure S3. Leave-one-out plots for the causal association between T2D and gut microbiota. Each panel corresponds to one microbial taxon and shows leave-one-out MR results, where the causal effect estimate is recalculated after removing one SNP instrument at a time (rsID listed on the y-axis). The black line indicates the leave-one-out point estimates, and horizontal lines represent 95% confidence intervals. The estimate using all included SNP instruments is shown at the bottom. Consistency of the leave-one-out estimates suggests that the MR result is not driven by a single SNP.
