# Supplementary material for: Gut Microbiota and Type 2 Diabetes: Genetic Associations, Biological Mechanisms, Drug Repurposing, and Diagnostic Modeling
Source: Int J Mol Sci. 2026 Jan 21;27(2):1070. doi: 10.3390/ijms27021070 (PMC12842411; doi:10.3390/ijms27021070)
Supplement: Supplementary file 1 [file ijms-27-01070-s001.zip › supplementary material_Figures/supplementary material_figS4.pdf]

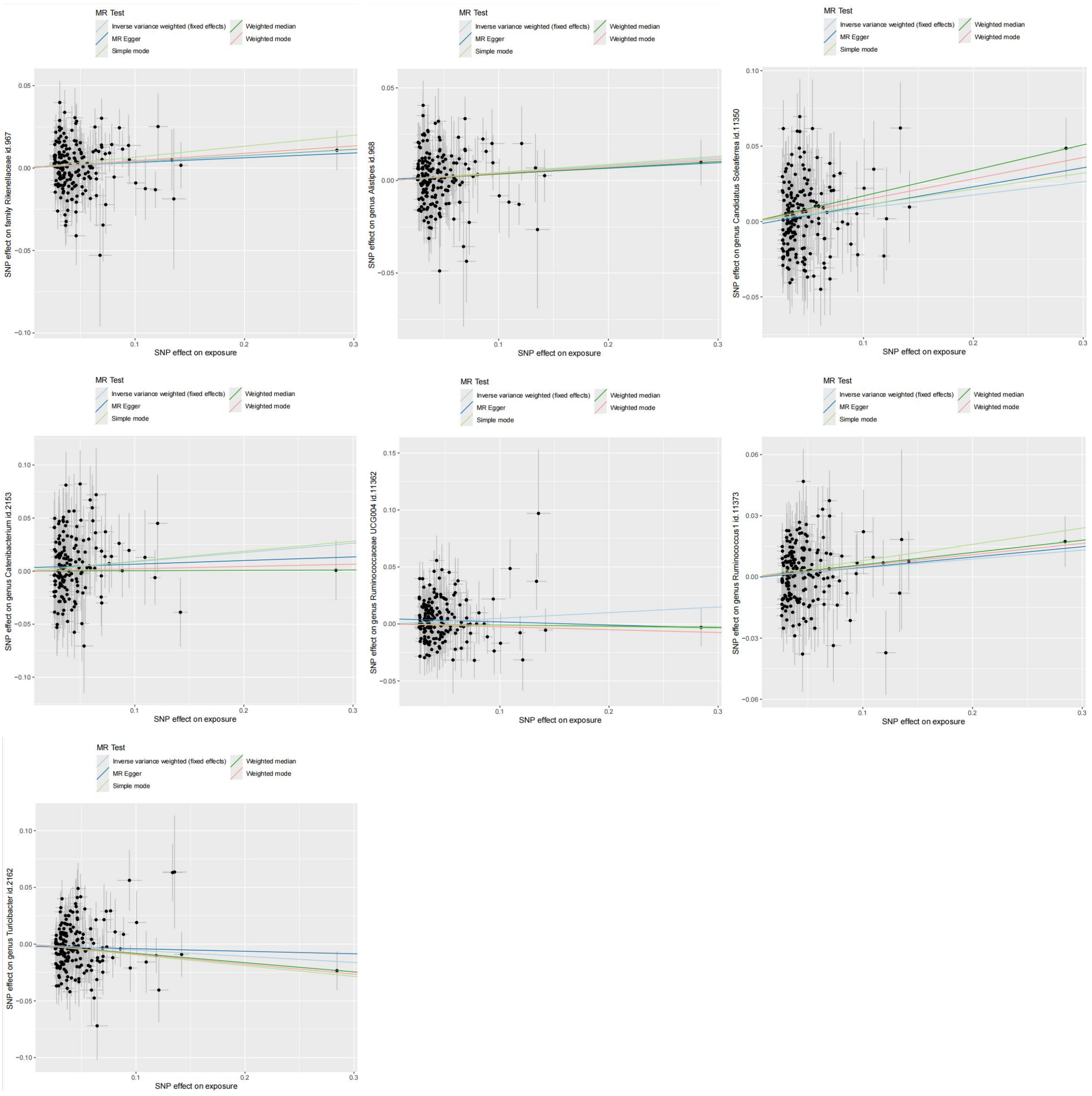

Figure S4. Scatter plots for the causal association between T2D and gut microbiota. Each panel corresponds to one microbial taxon (labeled on the y-axis) and plots the SNP–exposure association against the SNP–outcome association. Points represent individual SNP instruments, with horizontal and vertical error bars indicating the standard errors of the SNP effects on the exposure and outcome, respectively. The fitted lines show causal effect estimates from different MR methods (as labeled in each panel), including inverse-variance weighted (IVW; fixed-effects or multiplicative random-effects, as applicable), MR-Egger, weighted median, weighted mode, and simple mode. Consistency in direction and magnitude across methods supports the robustness of the inferred causal effect.
