# Supplementary material for: Gut Microbiota and Type 2 Diabetes: Genetic Associations, Biological Mechanisms, Drug Repurposing, and Diagnostic Modeling
Source: Int J Mol Sci. 2026 Jan 21;27(2):1070. doi: 10.3390/ijms27021070 (PMC12842411; doi:10.3390/ijms27021070)
Supplement: Supplementary file 1 [file ijms-27-01070-s001.zip › supplementary material_Figures/supplementary material_figS5.pdf]

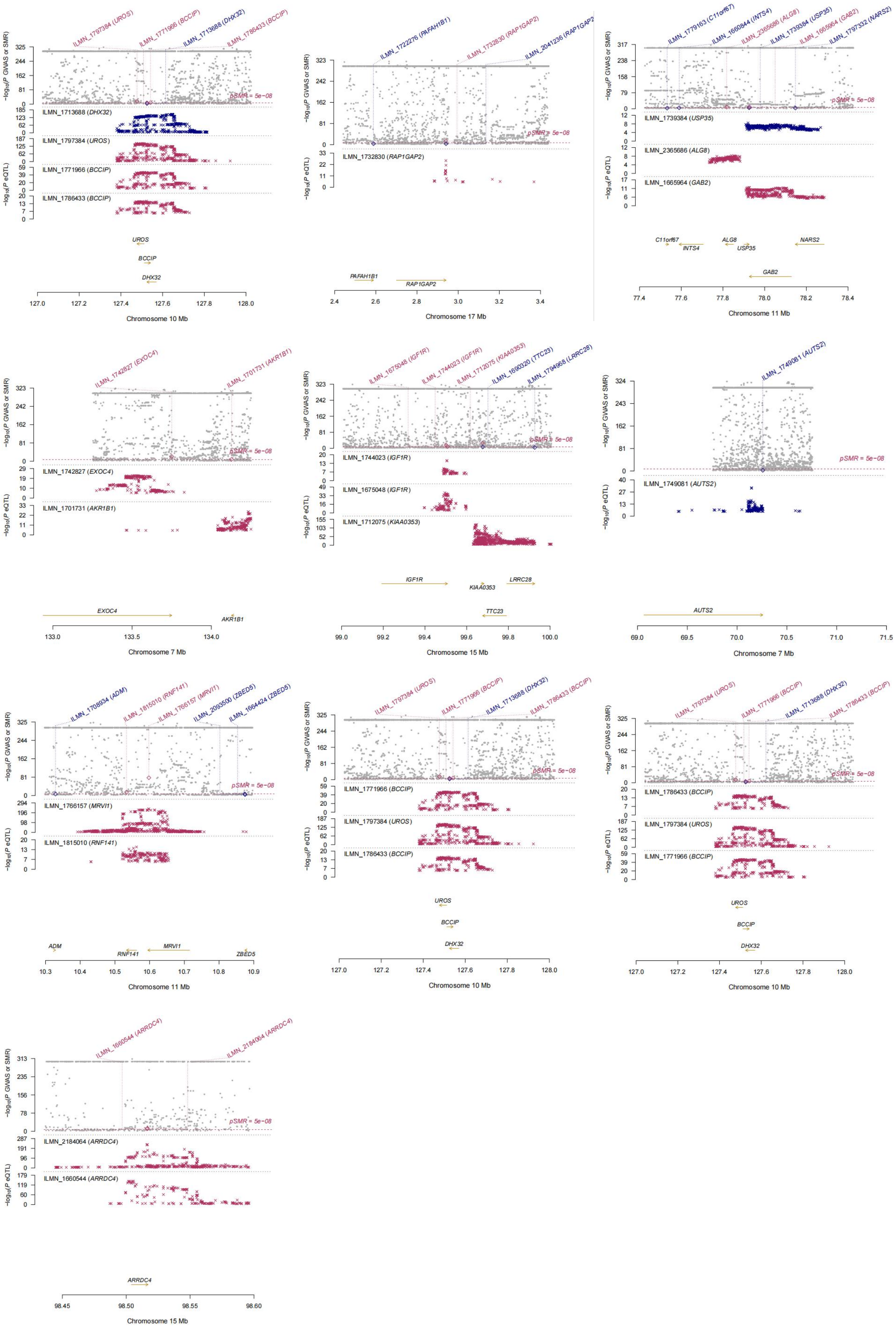

Figure S5. Regional association plots for SMR results. For each locus, the top track shows GWAS association signals for T2D (grey dots, $-\log_{10}P$) and SMR test results for expression probes (diamonds; plotted at the top cis−eQTL SNP for each probe). The middle tracks show cis−eQTL association signals for the corresponding expression probes (×, $-\log_{10}P$), with the SMR-prioritized probe highlighted (blue) and other probes shown in red, as labeled. The dashed horizontal line indicates the SMR significance threshold (pSMR = 5×10^{-8}), and the vertical dashed line marks the top cis–eQTL SNP used as the instrument. Gene annotations and genomic coordinates (Mb) are shown at the bottom.
